# Exploring Retention Among Army Nurses: A Qualitative Study

**DOI:** 10.1097/AJN.0000000000000220

**Published:** 2025-12-24

**Authors:** Stephanie K. Kessinger, Jacqueline Jones, Lori L. Trego

**Affiliations:** **Stephanie K. Kessinger** is a U.S. Army lieutenant colonel and chief of nursing science at Landstuhl Regional Medical Center, Landstuhl, Germany. **Jacqueline Jones** is a professor and assistant dean, and **Lori L. Trego** is a professor, both at the University of Colorado College of Nursing, Anschutz Medical Campus, Aurora. The authors have received funding from the TriService Nursing Research Program (Award No. 11207-N24-A05) at the Uniformed Service University, Bethesda, MD. The views expressed here are those of the authors and do not necessarily reflect the position or policy of the U.S. government, Department of Defense, Army Nurse Corps, TriService Nursing Research Program, or Landstuhl Regional Medical Center. Contact author: Stephanie K. Kessinger, stephanie.k.kessinger.mil@health.mil. The authors have disclosed no potential conflicts of interest, financial or otherwise.

**Keywords:** military nursing, nurse retention, qualitative research

## Abstract

**Background:**

Since the COVID-19 pandemic, the turnover rate among U.S. nurses has remained elevated, and now stands at 16.4%, costing the average hospital organization more than $3 million annually. The U.S. military faces similar challenges in its nursing workforce, with rising resignations and increasing job demands resulting in a shortage of more than 1,300 active-duty nurses. Without sufficient military nurses, service members, their families, and our national defense may be negatively impacted. We therefore felt it was important to examine how military nurses' experiences influence retention.

**Purpose:**

This interpretive, descriptive, qualitative study aimed to explore the unique experiences of active-duty U.S. Army nurses to determine the concepts that influence retention in the military.

**Methods:**

Twenty-four active-duty Army nurses with varying levels of nursing experience were recruited via social media. Semistructured interviews were conducted via Zoom. A six-phase cyclic process of reflexive thematic analysis was used to generate the findings.

**Results:**

The concepts that influence the retention of active-duty Army nurses are multifactorial. The main themes identified were (1) “*It's the people*,” consisting of leaders, mentors, the Army nurse community, and military care recipients, and (2) “*I'm not only a nurse*,” capturing the dual professional roles of nurses, who are also military officers, while also considering their nonprofessional roles as individuals and family members.

**Conclusions:**

Our findings suggest that positive interpersonal relationships and the ability to balance multiple professional and nonprofessional roles contribute to retention among active-duty Army nurses. Future retention efforts should include fostering relationship-based leadership styles, enhancing mentorship opportunities, uniting the military nursing community, and instituting a clinical career track so nurses can remain focused on their patient population and the nursing profession.

The turnover rate in U.S. nursing since the COVID-19 pandemic remains high, and is currently reported to be 16.4%, costing the average hospital organization more than $3 million annually.^1^ The U.S. military faces similar challenges in its nursing workforce, with rising resignations and increasing job demands resulting in a shortage of more than 1,300 active-duty nurses.^2^ The nursing shortage compounds the problem of retention. A higher workload for the remaining nurses can contribute to job dissatisfaction, burnout, and additional turnover.^3^ Nurse retention is, therefore, critical to alleviating the cyclic turnover pattern and positively affecting the overall functioning and cost-effectiveness of health care organizations.

Studies on improving nurse retention within the U.S. military report key contributors that are similar to those identified in the general U.S. nursing population, such as leadership,^4^ coworker relations,^5^ and the nursing practice environment.^6^ While these contributors are relevant to military nursing retention, the unique experiences of active-duty military nurses in particular have yet to be explored. This gap in understanding the concepts specifically influencing retention among active-duty nurses must be addressed to mitigate increasing shortages. Special consideration must be given to military nurses' function within two complex and intertwined social-ecological systems, the Military Health System and the U.S. armed forces.

The U.S. armed forces have approximately 16,000 military nurses serving in the U.S. Army, Navy, and Air Force Nurse Corps in active-duty, National Guard, or Reserve status.^7^ Military nurses have dual roles, encompassing their nursing and military officer responsibilities.^8^ They are one of the most deployed military specialties, as they respond to and support wartime missions, humanitarian efforts, and national emergencies.^7^ Without sufficient military nurses, service members, their families, and our national defense may suffer. It is therefore important to examine how their experiences, which include their dual professional roles as well as nonprofessional roles, influence retention.

**Study purpose**. This interpretive, descriptive, qualitative study aimed to explore the unique experiences of active-duty U.S. Army nurses to determine the concepts that influence retention in the military.

## METHODS

**Design**. We conducted an interpretive, descriptive, qualitative study to enhance our understanding of the concepts that influence military nurses' retention.^9^ Our team was composed of three investigators. The insider perspective of the principal investigator (one of us, SKK) as an active-duty Army nurse with 15 years of service was useful as we aimed to co-construct meaning with the study participants. Academic and military institutional review boards approved this study, and electronic consent was obtained from all participants.

**Sample and data collection**. Our purposeful sampling strategy captured variations in participants' experience, military rank, and sex to ensure different perspectives.^10^ Inclusion criteria consisted of nurses currently serving on active duty in the U.S. Army, excluding other military services (that is, Navy or Air Force nurses) and other Army components (that is, National Guard or Reserve). Recruitment flyers were posted on a closed-group social media page to reach Army nurses in different locations worldwide. Data were collected from September 2023 to April 2024 and included a demographic survey, semistructured interviews, field notes, and the principal investigator's reflexivity journal.

The demographic survey included an open-ended question to prepare the participants to discuss their reasons for being an Army nurse. Virtual semistructured interviews were conducted by the principal investigator. To establish rapport with participants, each interview began with a personalized question based on the participants' previous responses on the survey. Subsequent questions addressed topics guided by an ecological systems model.^11^ See Table 1,^11^ for the semistructured interview guide. Audio files were professionally transcribed, quality checked (by SKK), and deidentified. All data were stored in ATLAS.ti (version 24), a qualitative software program, for organization and assistance with analysis.

**Table 1.** A Semistructured Interview Guide

Guiding Ecological Level^a^	Questions Posed to Participants
Opening	First, please tell me why you pursued military nursing.
Individual	What drives you personally to continue to serve?
Microsystem	The next question is about your typical day-to-day routine at work and at home. Please describe your typical day.
Mesosystem/exosystem	I would like you to take a moment to reflect on the nested organizations surrounding your day-to-day. What, if any, organizational changes, directives, or decisions have influenced your decision to stay on active duty?
Macrosystem	Within Army nursing, you have two cultures with different values, roles, or norms. You have the nursing role and the military officer role. How do you balance these roles?
Chronosystem	Can you describe a pivotal event or series of events that shifted your decision to stay or leave the Army Nurse Corps?
Closing	If you could change anything about the Army Nurse Corps to make it better, what would you change?

a Ecological levels are based on the ecological systems theory.^11^

**Data analysis**. Reflexive thematic analysis guided the data analysis.^12^ This six-phase cyclic process (familiarization, coding, initial themes, theme development, refining themes, and write-up) began during the study design with self-reflection and lasted until manuscript production. This inductive analysis of coding, grouping, and development of initial themes was completed by the principal investigator. Weekly discussions with the research team led to the identification of patterns in the data and the development of thematic categories. Steps to ensure trustworthiness were taken by maintaining a meticulous audit trail, regularly sharing reflexive journaling among the team, clearly stating the principal investigator's position as a military nurse with every participant, and verifying interpretations with participants in the moment. Interpretative triangulation among both the military and civilian team members' perspectives was employed to monitor for blind spots.

## RESULTS

The final sample consisted of 24 active-duty U.S. Army nurse officers; 50% were men, with a mean (SD) age of 34.8 (10) years and military nurse experience of 9.9 (9) years. Nurses were junior ranking, company-grade officers (lieutenants and captains) and senior ranking, field-grade officers (majors and lieutenant colonels). The sample comprised a highly educated group (all had at least a bachelor's degree) of primarily White participants from diverse clinical specialties (most were medical–surgical, critical care, or perioperative nurses) and geographic locations (three were stationed in Germany, 21 throughout the United States). The average Zoom interview lasted 48 minutes; interviews ranged from 30 to 106 minutes.

Two main themes were generated from the data: (1) “*It's the people*” and (2) “*I'm not only a nurse*.” Each theme is described below, and is supported by participant quotes, field notes, and reflexivity journal entries. Participants are identified by the abbreviation AN for “Army nurse” and a number from 1 to 24.

**Theme 1: “*It's the people*.”** The designation of this theme was inspired by a response from AN18: “It's the people. That's why I stay.” Participants spoke of their relationships with others as the reason for staying, mentioning current or previous leaders; mentors and mentees; fellow Army nurses; and the unique patient population of military service members, their families, and veterans. Participants' experiences with people (both positive and negative) influenced their reasons for staying. Four concepts that were formed from the data were leadership, mentorship, community, and military patient care.

*Leadership*. This concept concerned the participants' interpersonal relationships with Army nurse leaders. Participants frequently spoke of their positive experiences with leaders as influencing their decision to remain an Army nurse. Based on these positive experiences, three leadership traits were extracted from the interview transcripts as important to retention: advocacy, empathy, and authenticity. AN17 cited these traits when describing his current nurse leader, who supported training opportunities while “prioritizing personal well-being, checking in with our family,” and “genuinely getting to know our concerns.” Similar sentiments were echoed throughout the dataset, as participants spoke of experiences with leaders who focused on them as people, not just task workers. In contrast, negative experiences with leaders made participants uncertain about staying in the Army Nurse Corps (ANC).

Leaders who advocated for participants were described as supportive and understanding, both professionally and personally. They were seen as supporting participants' professional growth as nurses and officers by promoting additional training and resources and by “putting our careers first” (AN8), while still realizing the daily mission of patient care. Additionally, these leaders advocated for participants' needs as they related to family and self. They were described as “fighting” (AN6), “protecting” (AN21), providing “top cover” (AN11), and going above and beyond to support participants. The recipients of this kind of leadership were grateful and appreciative, even when leaders (through no fault of their own) failed in their advocacy efforts.

Participants described empathetic leaders as influencing their decision to stay by displaying behaviors of self-awareness and caring. These self-aware leaders were described as “approachable” (AN23), providing feedback, and understanding how their actions and behaviors affected subordinates. This caring behavior was sometimes unexpected but greatly appreciated. AN3 recalled that when she was a lieutenant, she decided not to leave because “I had a good leader who cared about me and told me that I was good, which I think we don't do enough.”

In addition to advocacy and empathy, participants described their experience with authentic leaders who appeared genuine, sincere, and receptive. These positive experiences occurred with leaders at multiple levels, from the nurse manager to the ANC's highest executive leadership. The executive leaders of the ANC were often geographically distant from participants, nonetheless they were characterized by subordinate nurses as displaying genuine concern when discussing ongoing challenges (AN15). Additionally, participants mentioned feeling valued when leaders requested and listened to their input and expressed their appreciation (AN3, AN9).

*Mentorship*. Participants said that their role as a mentor or mentee had influenced their decision to stay in the military. They stated that serving as mentors for other Army nurses, medics, and cadets was the “best part of their job” (AN15) as it was “rewarding” (AN3), “fulfilling” (AN18), and a way “to give back” (AN21). As for receiving mentorship, both junior and senior ranks of Army nurses expressed gratitude for the guidance of other military officers. Junior officers noted their appreciation for senior ANC officers actively seeking out mentoring opportunities (AN7, AN8). Meanwhile, senior ranking Army nurses mentioned that their mentors often provided a fresh perspective and advice that influenced their decision to stay in the Army longer (AN4).

On the other hand, a lack of mentorship disappointed some participants, particularly junior officers (AN12, AN20). These company-grade officers desired more personalized feedback on career performance, opportunities, and career timing. Field notes further supported participants' desire for more mentorship opportunities, as 58% of company-grade participants requested to speak with the principal investigator after the interview about career pathways and professional opportunities.

*Community*. A sense of community, such as having a shared understanding, camaraderie and teamwork, and a desire to develop the next generation, also contributed to participants' desire to stay. Being surrounded by nurses with similar experiences aided in creating a sense of belonging and purpose. Army nurses felt they were not alone, as another nurse would be there to support them personally or professionally (AN12, AN16). Participants reflected on their first years and subsequent assignments as an Army nurse and identified their team as what made their work environment most enjoyable (AN1). Lastly, participants portrayed a continuous desire to develop future generations of Army nurses by enhancing the skills of junior Army nurses and cultivating better nurse leaders than those they had experienced.

*Military patient care*. Participants found fulfillment and value in caring for others in the military, including service members, their families, and veterans at home or abroad. These Army nurses gained personal satisfaction in caring for multiple generations of service members and their families (AN7, AN12). They expressed a desire to care for service members, especially during overseas deployments and combat operations. One considered leaving the military early in his career, yet he stayed to care for combat-wounded patients during surges of the Iraq and Afghanistan wars (AN14). There was mutual appreciation, as the military patient population regularly expressed gratitude for the health care provided. The magnitude of the nurse's role in protecting our nation by maintaining the health and well-being of all service members was noted. Quotes from participants and the principal investigator's reflexivity journal supporting the influence of positive interpersonal relationships on the retention of Army nurses can be found in Table 2.

**Table 2.** Additional Quotes Related to Theme 1: “It's the People”

Reason for Retention	Quote	Source
Leadership		
Advocacy	“Leaders help set us up for success, like sending us to school and getting unique training opportunities.” “Good bosses make me come out of my comfort zone. They see me and they let me have a voice and grow.”	AN17 AN11
Empathy	“I wanted to get out when I had a bad leader. I felt nobody cared about me as a human being, and I needed to care for my family and myself.” “I had a great leader, he was cognizant that if he was in the office, his staff would be in the office. So, he made a point to leave work at 1700 every day. He set a good example of understanding the needs of your staff and subordinates.”	AN3 AN13
Authenticity	“She was a great leader because she was genuine, you could tell she really cared, and she was straight with all of us.” “I see genuine concern when talking with senior ANC leaders.”	AN8 AN15
Mentorship		
Mentors	“Having him as a mentor allowed me to see the value that I add to the Army.” “One of my mentors made me remember why I joined the military in the first place; it gave me purpose to continue to serve beyond my initial contract.”	AN13 AN6
Mentees	“After a year of training, I see her throwing in a chest tube without even hesitating. You'd think she had been doing it for 30 years. That kind of stuff speaks to me.” “The people are rewarding. It is totally worth it when a junior officer comes by and is like ‘that was so helpful’ or ‘you get me, ma'am’.”	AN18 AN9
Community		
Shared understanding	“When you PCS [relocate], you have a built-in group of friends. People who understand your life, and you don't have to explain yourself.” “To stay beyond those four years, it was feeling like I was part of a team and feeling like we had a common goal, albeit slow at times, I always felt like the team was moving forward.”	AN12 AN1
Camaraderie	“The overwhelming response to my first recruitment post was so supportive. It makes me think how this community of nurses is more than just coworkers. Do they want to see me succeed, or is it camaraderie?” “The camaraderie, teamwork aspect, that family cohesion that we get in the military with each other, is something I don't see on the civilian side.”	Reflexivity journal AN19
Next generation	“What influenced me to stay is my ability to enable lieutenants. I want to give them all the things I wish I had known, and giving back to the next generation is what I want to keep doing.”	AN24
Military patient care		
Service members	“The best part about being an Army nurse is taking care of soldiers, from D-day veterans to recently enlisted soldiers, and seeing the whole spectrum that everyone's a soldier at the end of the day.”	AN7
Families	“Maybe not everybody views it as such, but we know how important it can be to that person downrange that we are caring for somebody's family member.”	AN12
Combat care	“There was a lot of competition among my peer group to get deployed because that's what we came in for.” “Our national security is based on our soldiers being able to focus and be healthy, their families being healthy, and them being able to do what they need to do. Our job ensures safety.”	AN11 AN12

**Theme 2: “*I'm not only a nurse*.”** This theme highlighted the multiple roles of Army nurses. As AN12 stated, “You're not only a nurse, you're an officer and a leader.” We interpreted and categorized Army nurse roles into professional and nonprofessional. The professional roles include the nurse and officer (soldier and leader), while the nonprofessional roles include the family and self. Each participant's ability to balance these roles influenced their decision to continue serving as an Army nurse.

*Professional roles: nurse and officer*. As both health care providers and officers, Army nurses have experiences that involve a complex dyadic interaction. The challenges of their dual roles were interpreted from both a systems and an individual perspective. As officers, Army nurses (like other soldiers) have unique requirements that are not typically expected of civilian nurses, such as having to enroll in military courses, pass physical fitness tests, appear before promotion boards, undergo frequent relocations, and maintain deployment readiness. These Army requirements were mentioned by participants as a source of stress and anxiety, especially when they're also juggling nursing roles and responsibilities. Participants often described additional tension when working within two complex systems: the health care system (Military Health System) and the military system (Department of the Army). One participant described the health care system as leveraging nurses' moral duty to care for patients, even when it caused burnout, because nurses knew if they didn't do it, they would “be replaced by someone else [another Army nurse]” who would have to. Participants were pulled in different directions, torn between their two professional roles, as each system (health care and military) had different priorities.

Participants described their personal preference—either spending more time as a nurse or as an officer—as important to them. Frustrations arose when they could not focus on their preferred role, especially if their desire to focus on patient care was dismissed and they were forced into nonclinical jobs. Furthermore, some participants felt that their nursing skills did not matter for career progression, since “terrible nurses” (AN16) were selected for promotion to a higher rank. However, other participants described their professional preferences as aligning more closely with the military due to the varied positions and assignments available. The ability to change jobs every few years enticed many participants to continue in the military, as new experiences came with new opportunities. Yet, the inability to relocate or change jobs more frequently created feelings of being “held back” (AN12) or “pigeonholed” (AN23).

The benefits of serving as an Army nurse were also acknowledged and described as encompassing both monetary and nonmonetary opportunities and rewards. Educational opportunities included fully funded specialized training and time to pursue advanced degrees while continuing to serve on active duty. Various job opportunities, such as the ability to switch between clinical and nonclinical positions, were another reason participants continued to serve. Other benefits, such as health care coverage, retirement accounts, retention bonuses, and nontaxable allowances, were also mentioned as financial incentives to stay. Conversely, some participants described a desire to leave when these opportunities or benefits were removed or blocked.

*Nonprofessional roles: family and self*. Participants' experiences as both an individual and a family member contributed to their retention in the ANC. Each participant discussed their role and responsibility as a family member, spouse, parent, child, and/or pet owner. Despite their desire to remain an Army nurse because of their positive interpersonal experiences and professional balance, many felt the ability to serve was impossible without family support. AN21 commented on how “everything revolves around my family,” and that she nearly walked away from her career when faced with childcare challenges. Other female participants reported similar frustrations with finding reliable childcare and the need to depend on family in order to remain an Army nurse.

Participants described how their personality traits, beliefs, and values influenced their reasons to stay. Personality traits related to retention focused on seeking challenges, loyalty, and pride. Participants also noted their beliefs and values as other reasons to remain, as staying gave them a “sense of purpose” (AN19) or duty. Table 3 contains selected participant quotes regarding retention and the need to balance professional and nonprofessional roles in order to stay.

**Table 3.** Additional Quotes Related to Theme 2: “I'm Not Only a Nurse”

Reason for Retention	Quote	Source
Professional roles		
Career opportunities	“It's the opportunities that we are allotted and the places we can go, that's what drove me to stay.” “As a Nurse Corps officer, the opportunities to pursue education and professional growth is mind blowing. That's why I continue to be in.”	AN20 AN21
Dual role challenges	“They say we're officers first, but it doesn't show when you are doing your job because then you're a nurse first.” “You gotta have a balance of Army stuff and nursing stuff, it cannot be just nursing, nursing, nursing.”	AN23 AN8
Nonprofessional roles		
Family	“I'll stay past retirement eligibility if there's some opportunity that works well for our family at that time.” “I want to stay, but a few things could pull me out of the Army Nurse Corps, one revolves around custodial rights to my children. I may need to stay here.”	AN9 AN2
Self	“Staying because it challenges yourself in ways that the civilian world cannot.” “My belief system and faith run hand in hand with Army values.”	AN6 AN19

## DISCUSSION

Given the current rate of nurse turnover, nurse retention is highly desirable. Understanding the concepts influencing retention in this sample of military nurses can assist health care leaders seeking to improve retention within their organization. This study found that interpersonal relationships contributed to retention, including relationships between nurses and leaders, mentors, other members of the ANC community, and the unique military patient population. Furthermore, Army nurse retention requires balancing professional and nonprofessional roles, including being a nurse, a soldier, a family member, and an individual. These concepts of leadership, mentorship, community, patients, nursing, military, family, and self contribute in various ways to Army nurse retention.

Leadership and mentorship are known factors that influence nurse retention among both civilian^13^,^14^ and military nurses.^4^,^15^ Relationship-based leadership styles and enhanced mentorship opportunities play key roles in encouraging future development and retention. Our study's sample included mostly millennial nurses (that is, nurses born between 1981 and 1996). A recent study conducted among members of this same age group found that encouraging supportive and open communication with nurse leaders and fostering professional growth through mentorship are effective retention strategies.^13^

Our findings are also consistent with other research showing that nurses demonstrate a greater intent to stay when satisfied with coworker relationships.^5^,^14^ We noted extensive loyalty among Army nurses as one reason for retention. Camaraderie has been shown to contribute to retention among other health care professionals, including both military^16^ and civilian medical personnel.^17^,^18^ Future solutions must consider ways to support the nursing community, especially during pandemics or other major catastrophic events.

Other studies have examined specific work demands or patient populations and job satisfaction among nurses in the armed services.^19^-^21^ In our study, the opportunity to provide nursing care to the military patient population, specifically to deployed service members, was particularly meaningful and impacted retention among Army nurses. Participants' expressions of gratitude and expectation to care for the health and well-being of service members in any setting align with the Army values of duty and selfless service. Future retention strategies should consider providing opportunities for nurses to care for the patient population that reflects their desires and personal values.

Participants described the benefits and challenges of serving in two professional roles. The benefits included opportunities to serve in various positions, including nonclinical roles, such as those involving recruiting and command. We found, like others did, that perceived opportunities were a motivation to stay in the military^21^ and in other military medical professions.^22^ Our findings also indicate that Army nurses face personal challenges when attempting to balance their professional roles, as their desire to remain closer to the nursing role was overshadowed by more officer-focused roles. Future initiatives may consider instituting a clinical career track for those who prefer to remain involved in the nursing profession, while also developing a way to foster professional and promotional growth and opportunities.

Nonprofessional roles related to family and an individual's personality traits, beliefs, and values also influenced Army nurse retention. In a previous study, family-related factors were identified as contributing to military nurses' decisions to remain in or leave the service; notably, frequent family relocations were shown to have a negative impact on retention.^21^ Healthy work environments that support work–life balance may improve nurse retention.^13^ Leaders must continue to recognize and consider the importance of nonprofessional roles and provide flexible work schedules when possible.

**Limitations**. There are a few limitations in this study regarding participant sampling that may have had an impact on the findings. Our purposeful sampling focused on rank and sex but failed to account for the current job assigned. A review of the participants' comments revealed that only 33% of participants were primarily working in a direct patient care position. Limited perspectives from direct patient care nurses could have impacted the overall findings. An additional limitation relates to our social media recruitment. The recruitment flyer was posted on a closed Facebook group of Army nurses with the word “mentorship” in the title. This might have attracted participants who were actively seeking mentorship, as reflected in our results. Lastly, while this study focused on retention, sampling criteria excluded Army nurses who had recently left the military. Including these nurses may have provided further insight into their reasons for leaving.

## CONCLUSIONS

This study highlights the importance of positive interpersonal relationships and the ability to balance professional and nonprofessional roles as contributors to Army nurse retention. In the future, military nurse leaders and researchers should consider fostering relationship-based leadership styles, enhancing mentorship opportunities, uniting the military nursing community, and providing more avenues to care for service members in a variety of settings. Our study results provide valuable insight into concepts influencing Army nurse retention, such as the importance of nurse leaders demonstrating advocacy, empathy, and authenticity; mentorship opportunities; a connected nursing community and camaraderie; and caring for a specific patient population. In addition, Army nurses' retention is influenced by their ability to juggle multiple roles. These include the dual professional roles of nurse and officer and the nonprofessional roles of self and family, as well as personality traits, beliefs, and values. Ensuring that these concepts are addressed in present health care organizations should improve nurse retention for the health and well-being of nurses and patients alike.
